# Unobstructed orthopaedic surgical robot assisted percutaneous iliosacral screw fixation of sacral brittle fractures

**DOI:** 10.3389/fmed.2023.1218720

**Published:** 2023-11-16

**Authors:** Xiao-dong Hao, Yuan-zhi Zhang, Shao-bai Wang, Gang Liu

**Affiliations:** ^1^Department of Orthopaedics, The Affiliated Hospital of Inner Mongolia Medical University, Hohhot, China; ^2^School of Kinesiology, Shanghai University of Sport, Shanghai, China

**Keywords:** bone screws, fracture fixation, sacrum, robot, sacroiliac joint

## Abstract

Pelvic fractures mostly result from high-energy injuries in life; the longitudinal fracture of the sacrum is the most common type of sacrum fracture. This study was designed to evaluate the accuracy, safety, and efficacy of percutaneous sacroiliac joint screw placement in the treatment of longitudinal sacrum fractures with the assistance of unobstructed orthopaedic surgery robots. According to different surgical methods, 32 patients were divided into robot group and free hand group, with 16 patients in each group. The operation time, intra-operative blood loss, intra-operative fluoroscopy times, screw placement angle deviation were collected. There were statistically significant differences in terms of angle deviation of screw placement (1.96 ± 0.75° vs. 2.87 ± 1.03°; *p* = 0.0145), deviation of the guide needle (1.92 ± 0.93 mm vs. 2.91 ± 1.22 mm; *p* = 0.0209), intra-operative fluoroscopy time (7.25 ± 1.72 s vs. 20.93 ± 5.64 s; *p* = 0.0000), insertion time of each sacroiliac joint screw (14.72 ± 2.66 min vs. 29.21 ± 5.18 min; *p* = 0.0000). There was no statistically significant difference in terms of blood loss (100.21 ± 7.37 mL vs. 102.52 ± 8.15 mL; *p* = 0.4136). These results suggest that orthopaedic surgery robot for the treatment of longitudinal sacrum fracture is safer and provides less irradiation than the traditional freehand methods.

## 1. Introduction

Pelvic fractures mostly result from high-energy injuries in life, accounting for a large proportion of all systemic fractures, about 3% (1–3). Sacral brittle fracture is a common and special type of brittle pelvic fracture. Normal daily stress repeatedly concentrates on the osteoporotic sacrum, resulting in a brittle fracture of the sacrum called sacral incompetence fracture. It has the characteristics of high missed diagnosis rate and easy re-displacement of fractures. Due to the degenerative changes of the pelvis in the elderly, it is often manifested as bone resorption on both sides of the sacroiliac joint and pubic symphysis, calcification of the ligaments around the joint, and narrowing of the joint space. Due to special anatomical characteristics and low energy lateral compression, brittle pelvic fractures often manifest as posterior sacral wing compression fractures and pubic ramus fractures on both sides of the anterior pubic symphysis. In recent years, sacroiliac screws, sacroiliac vertebroplasty, and bone cement reinforced sacroiliac screws can significantly alleviate pain and improve mobility, making them effective minimally invasive treatment techniques (4, 5). The longitudinal fracture of the sacrum is the most common type of sacrum fracture. In the traditional treatment methods, open reduction and internal fixation are often used to treat this injury (6, 7), or S1 vertebral fixation is done by transverse screw placement in the sacroiliac joint to stabilize the sacrum fracture, so as to ensure the stability of the posterior pelvic ring. Studies abroad have shown that the invasiveness and complications of longitudinal sacrum fractures are significantly reduced by transversely placing screws in the sacroiliac joint to fix S1 vertebrae. And the treatment of sacrum fractures by screw fixation in the posterior pelvic sacroiliac joint has a very reliable mechanical strength and can provide consistent pelvic stability (8, 9).

However, due to the complexity of the anatomy around the sacrum, the placement of sacroiliac joint screws is prone to place the screw at an unsatisfactory angle and length during the operation and to break the screw during the operation if the screw is placed many times (10). The technology of sacroiliac joint transverse screw placement under computer navigation effectively reduces the difficulty of surgery. By planning the best screw channel before surgery, the safety and accuracy of screw placement are improved (11). However, there is also the problem of long exposure time of intra-operative rays, and due to the complicated structure in the pelvis and the deep location, this treatment requires high accuracy in screw implantation (12). The application of surgical robots makes up for the lack of manual operation of the surgeon. It can complete the same task repeatedly and accurately, reduce operation time, improve operation accuracy, and ensure the safety of doctors and patients (13, 14). Therefore, on this basis, the concept of sacroiliac joint screw implantation for the treatment of longitudinal sacrum fractures with the assistance of orthopaedic surgical robots is introduced (15). The goal of the study was to evaluate the accuracy and safety of sacroiliac joint screw implantation assisted by the robot in the treatment of sacrum fracture.

## 2. Materials and methods

### 2.1. Study design

A retrospective analysis of the clinical data of 32 patients with sacrum fractures were admitted to the Affiliated Hospital of Inner Mongolia Medical University from September 2018 to December 2020. This study was approved by the Ethics Committee of Inner Mongolia Medical University Affiliated Hospital, and obtained the informed consent of all the subjects.

### 2.2. Inclusion criteria

Patients were included according to the following criteria: (1) Closed and unstable sacral fractures, with or without fracture displacement, can be treated with closed reduction (or limited open reduction), after which the sacroiliac joint has space for cannulated screw placement; and (2) An open, fixable sacrum fracture can be reduced to a closed fracture after initial treatment and can be reduced by reduction and screw placement.

### 2.3. Exclusion criteria

Exclusion criteria were: (1) Severe open injuries or rupture of abdominal cavity and organs with wound contamination; (2) The unavoidable blood vessels and nerves and other tissues in the trajectory planned by the robot assistant system, or patients whose sacroiliac joint do not have space for the hollow screw placement after reduction, or the patients who are unable to use screws to fix sacral fractures effectively; and (3) Patients with systemic diseases, such as severe haemorrhagic diseases, severe heart, and respiratory diseases, or peripheral skin or soft tissue infections.

### 2.4. Patients and clinical variables

Thirty-two patients with longitudinal sacral fracture were divided into two groups: the robot-assisted group (AIOOR, All-in-one orthopaedic robot, Shanghai Zhuoxin Medical Technology Co., Ltd., China) and the freehand group. The robot-assisted group was treated with transverse sacroiliac joint screw implantation through the orthopaedic surgery robot assistant system, while the freehand group was treated with sacroiliac joint screw implantation by orthopaedic surgeons.

### 2.5. Surgical indications, procedures, and postoperative management

Both operations were performed by the same group of senior doctors.

#### 2.5.1. Conventional treatment

All patients undergoing surgery underwent routine admission tests after admission. Temporary pelvic external fixation was used for patients with hemodynamic instability. Patients with vertical instability of pelvic fracture were treated with tibial tubercle traction of lower extremities. All patients underwent CT scanning. On the premise of ensuring these patients’ hemodynamic stability, patients with vertical instability of pelvic fractures were examined regularly by X-ray to observe the reduction effect of their lower limbs.

##### 2.5.1.1. Robot system

**Figure 1:**
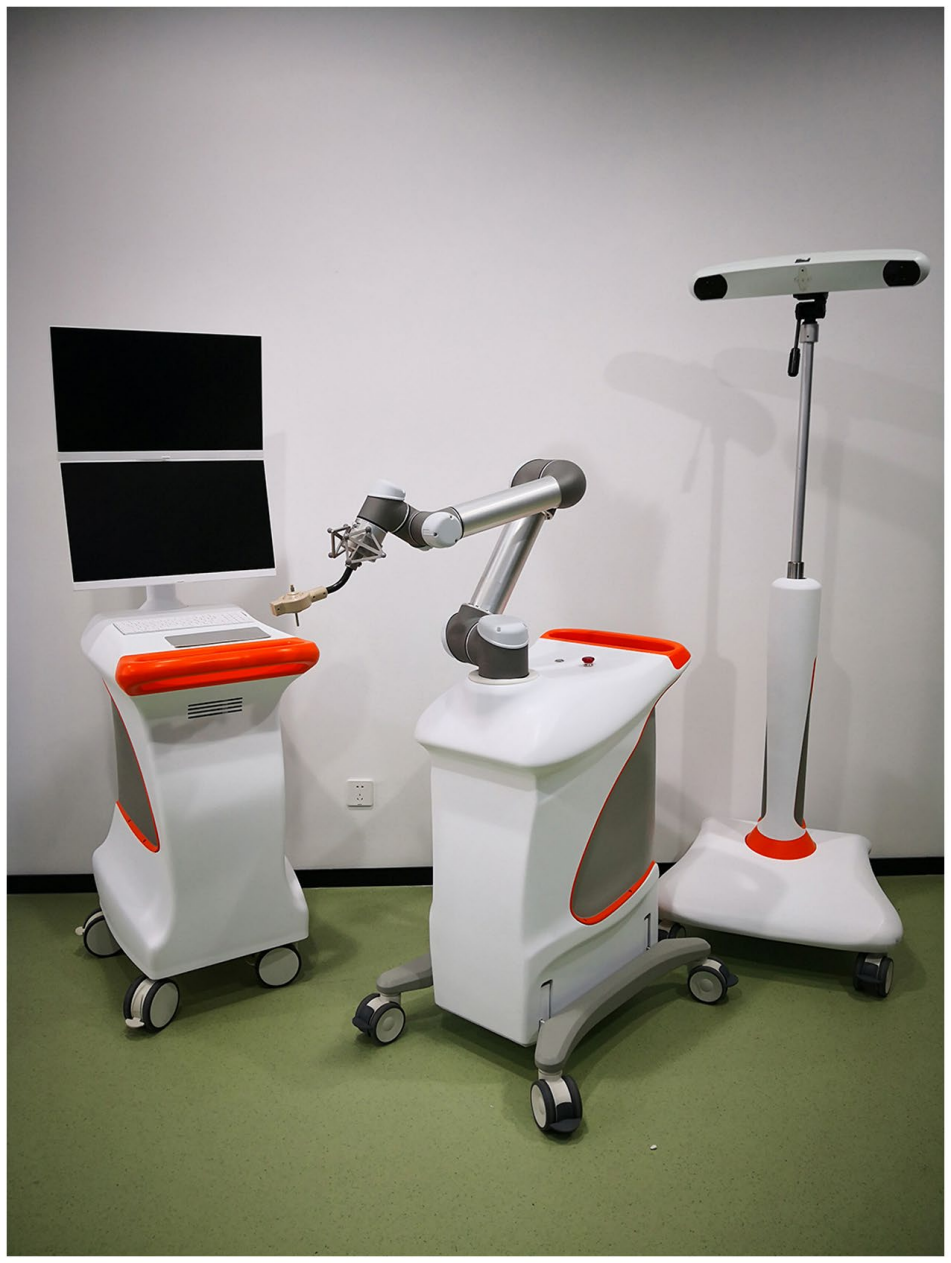
All-in-one orthopaedic robot.

AIOOR is a robot assistant system based on digital and minimally invasive surgery concept. The main body comprises a 6-degree-of-freedom manipulator, the main control trolley, and an optical tracking system (Figure 1).

#### 2.5.2. Surgical procedures

The patients were placed in a supine position. For patients with pubic ramus fracture, open reduction and internal fixation were performed on the pubic ramus after sacral reduction.

##### 2.5.2.1. Robot-assisted groups

**Figure 2:**
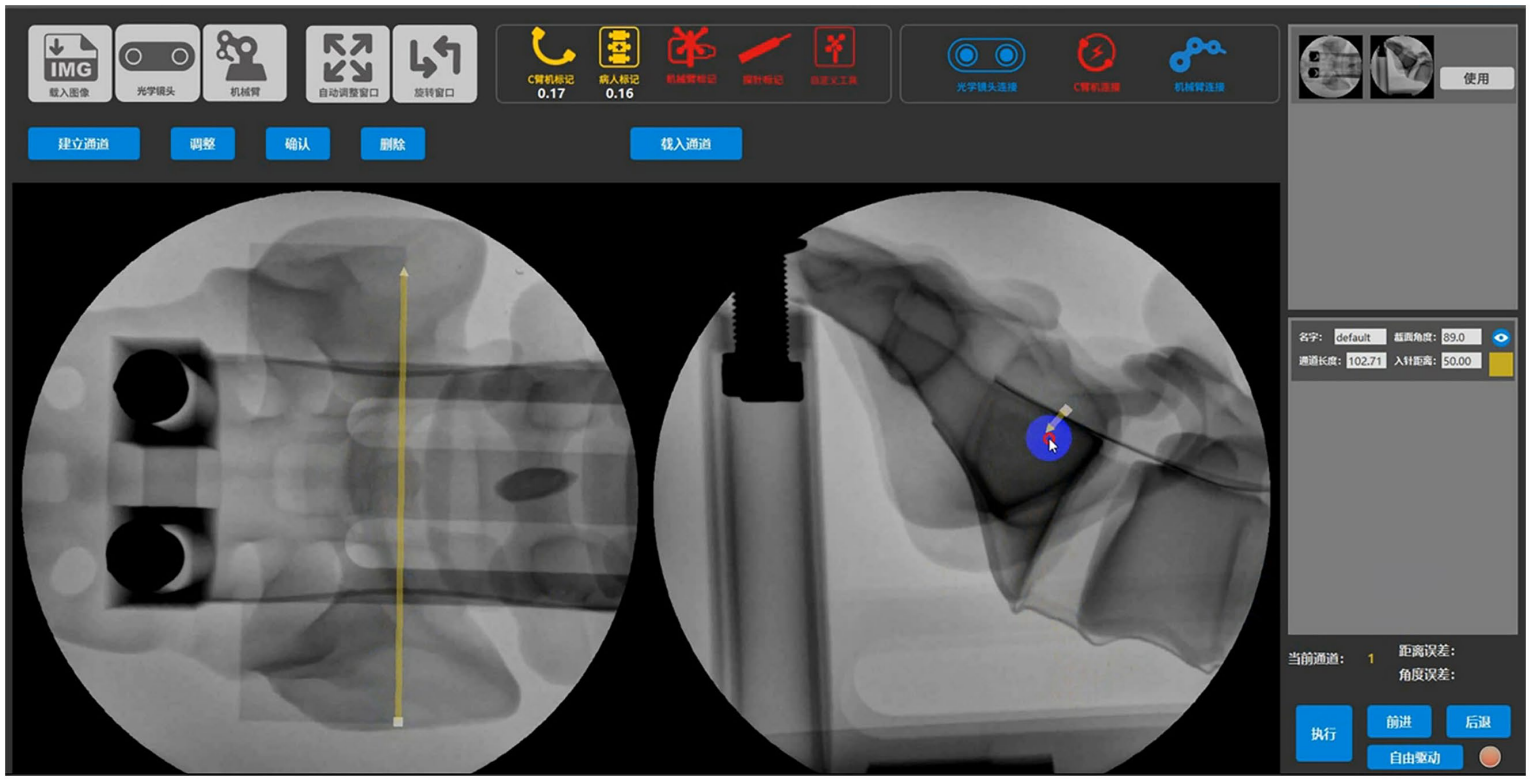
Determination of the best screw placement channel in the software (demo).

An entry point that was determined on lateral sacral and antero-posterior views during intrao-perative fluoroscopic X-rays (16, 17). With the robot-assisted procedure, the preoperative planning and establishing the optimal screw channel to ensure the channel was located in the safety zone of S1 vertebra (18, 19) (Figure 2). Optical trackers were first fixed on the patients, and then an optical tracker was fixed on robot. The spatial position of the overall operation trajectory of the manipulator during this sacroiliac joint screw placement operation was obtained through the bi-planar positioning algorithm of the orthopaedic surgery robot assistant system (20). When the manipulator completed the planned trajectory pass, the manipulator was locked in its current position. A 2.5 mm Kirschner wire was inserted into the pelvic bone throughing the sleeve. A 7.3 mm cannulated screw was inserted.

##### 2.5.2.2. Freehand group (under C-arm fluoroscopy with freehand conventional techniques)

According to the pelvic images obtained by C-arm fluoroscopy and the planned position of the screw pathway, a 2.5 mm Kirschner wire was inserted into the pelvic bone. Then, a 7.3 mm cannulated screw was inserted and fixed along the Kirschner wire.

#### 2.5.3. Postoperative management

The patients received pelvic X-ray, CT scan, and 3D imaging reconstruction to confirm the precision of the position of the ilio-sacral screw. The deviation between the actual and the planned optimal screw position was measured. The measurement method is as follows: The pelvis was scanned using a spiral CT scanner (Light Speed 64; GE, Boston, MA, United States) at 120 kV with a slice thickness of 0.625 mm and a matrix of 512 × 512 pixels. The generated images were converted into DICOM format and were further processed using medical imaging software (Mimics Innovation Suite 15.0; Materialise, Leuven, Belgium) to obtain the STL format files for the 3D reconstruction of the pelvises. Visualization software (Imageware 12.0; EDS, Plano, TX, United States) was subsequently used to produce multi-slice sagittal views (thickness of 1.0 mm) of the 3D reconstruction images. First, we defined the geometric boundary of the safe zone on each sagittal view for the first sacral vertebra. Thus, the inscribed ellipse of the boundary was obtained from each view. Subsequently, the X, Y, and Z coordinates of the center in each inscribed ellipse (i.e., the intersection of the major and minor axes) were calculated. Finally, the least-squares methods were used to fit the optimal axis that pass through the centers of the inscribed ellipses (a statistical procedure to find the best fit for a set of data points by minimizing the sum of the offsets or residuals of points from the line). This axis was defined as the optimal and safe pathway for the ilio-sacral screw. Also, the CT scan data obtained after surgery (the same parameters as before) were imported into the Mimics Innovation Suite for 3D reconstruction, and the actual screw position was compared with the optimal pathway in the software to measure the angle deviation of screws.

All patients received regular postoperative ambulatory follow-ups.

#### 2.5.4. Outcomes

According to postoperative imaging data and follow-up results, the overall clinical efficacy of sacroiliac joint screw placement in the treatment of longitudinal sacrum fracture was evaluated and scored according to the Majeed function system (21). The following indexes were compared in two groups of patients: (1) the time of intraoperative screw placement; (2) the number of X-ray exposure; (3) the blood loss; and (4) the deviation of the angle between the actual screw channel and the planned optimal screw channel.

### 2.6. Statistics

All the data were statistically analyzed by SPSS statistical software (version 13.0; SPSS, Chicago, IL, United States). The Shapiro–Wilk test was used to determine whether the data were normally distributed, and the measurement data were expressed as mean ± standard deviation. For the measurement data meeting the normality, the comparison between the two groups of patients was conducted by two-independent sample *t* test, and the comparison of measurement data at different time points in the same group was conducted by repeated measurement ANOVA. LSD *t* test was used for multiple comparisons, and non-parametric test was used for inter-group or intra-group comparisons for measurement data that did not meet the normal distribution. *p* < 0.05 was considered statistically significant.

## 3. Results

**Figure 3:**
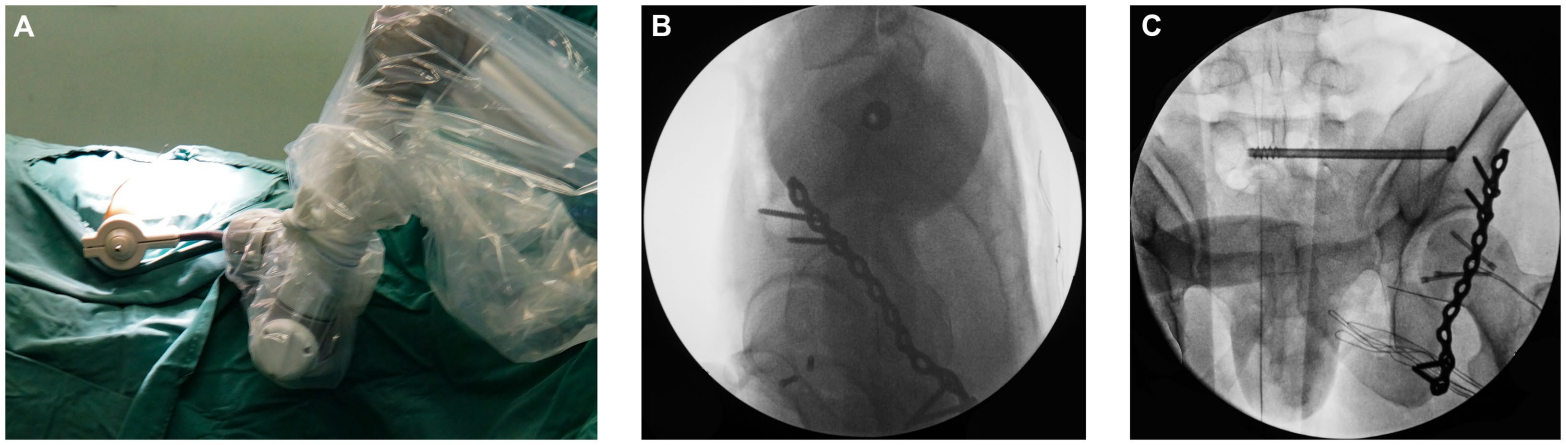
Surgical procedures with robot system: position of robot arm **(A)**, showing the guide sleeve and the channel **(B)**, placing the screw **(C)**.

**Figure 4:**
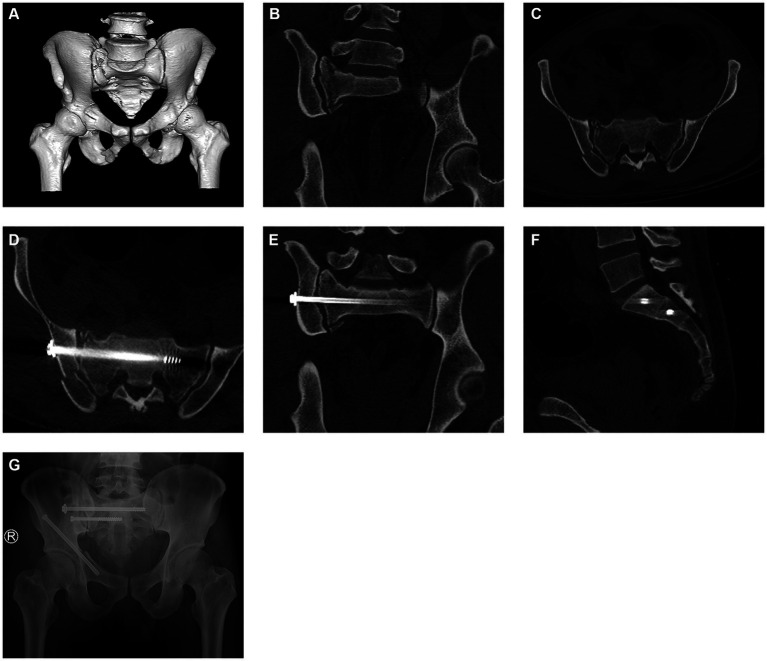
Postoperative images of the ilio-sacral screw assisted by robot. Fracture of sacrum **(A–C)**, Position of ilio-sacral screw **(D–F)**, Radiographs 6 months after surgery **(G)**.

32 patients with pelvic fractures were included in this study, including 23 males and 9 females. The patients’ age range was 62 to 76 years old, and the average age was 63.2 years old. Among them, 19 cases were injured in traffic accidents, 8 cases were injured by falling from a height, and 5 cases were injured by heavy objects. According to DENIS classification of fractures, there were 23 cases of type I fracture and 9 cases of type II fracture. Concomitant injuries included hemorrhagic shock (*n* = 5), rib fracture (*n* = 9), spleen rupture (*n* = 4), urethral rupture (*n* = 2) and limb fracture (*n* = 2) (Table 1). There was no damage to blood vessels and nerves caused by the deviation of sacroiliac joint screws or the sacrum canal invasion. All patients had 3 to 12 months follow-up after the operation. There were statistically significant differences in terms of average angle deviation of screw placement (1.96 ± 0.75° vs. 2.87 ± 1.03°; *p* = 0.0145), deviation of the guide needle (1.92 ± 0.93 mm vs. 2.91 ± 1.22 mm; *p* = 0.0209), intra-operative fluoroscopy time (7.25 ± 1.72 s vs. 20.93 ± 5.64 s; *p* < 0.001), insertion time of each sacroiliac joint screw (14.72 ± 2.66 min vs. 29.21 ± 5.18 min; *p* < 0.001). There was no statistically significant difference in terms of blood loss (100.21 ± 7.37 mL vs. 102.52 ± 8.15 mL; *p* = 0.4136). According to the Majeed function system, 22 patients achieved excellent scores, 7 patients got good scores, and 3 patients received acceptable scores (Figures 3, 4 and Table 2). The 16 patients in Robot assisted group had Majeed scores of over 85, and the excellent and good rate reached 100%. There were no complications such as additional surgery and screw loosening in the two groups.

**Table 1:** Patients’ characteristics of the two groups ($\bar{x}$ ± s).

Patient characteristics	Robot assisted group (*n* = 16)	Freehand group (*n* = 16)	*p* value
Age (years)	62.31 ± 5.92	63.93 ± 6.81	0.4837
Male	12	11	
Female	4	5	
BMI (kg/m^2^)	28.73 ± 0.78	28.19 ± 0.93	0.0954
Injury mechanism			
Fall	3	5	
Motor	11	8	
Crash	2	3	
Denis classification of fractures			
Type I	11	12	
Type II	5	4	

**Table 2:** Comparison of postoperative parameters between the two groups ($\bar{x}$ ± *s*).

	Robot assisted group	Freehand group	*p* value
Screw placement angle deviation (degree)	1.96 ± 0.75	2.84 ± 1.03	0.0145
Needle insertion deviation (mm)	1.92 ± 0.93	2.91 ± 1.22	0.0209
Intra-operative fluoroscopy time (s)	7.25 ± 1.72	20.93 ± 5.64	0.0000
Each sacroiliac screw insert time (min)	14.72 ± 2.66	29.22 ± 5.18	0.0000
Intra-operative blood (mL)	100.21 ± 7.37	102.52 ± 8.15	0.4136

## 4. Discussion

Sacroiliac joint screw placement technique fixes sacrum fractures by placing screws laterally in the sacroiliac joint to fix the S1 vertebral body. This is a central fixation method, which is more conducive to the stability of internal fixation in terms of biomechanics (22) and allows the patient to carry weight early (23). It also has the advantages of less trauma, less intraoperative blood loss, and less damage to the periosteum, playing a positive role in the postoperative recovery of patients (24). However, the sacroiliac joint screw placement technique also has its limitations. Due to the sacrum’s irregular structure, the passage of the sacroiliac screw is narrow and adjacent to the sacral canal. It is hard to control the direction of the screw during the screw placement process. Surgeons and patients will suffer a significant increase in radiation exposure time and operation time due to multiple C-arm adjustments to determine the relative spatial relationship between the screw and the sacroiliac joint (25). The route of screw placement cannot be fully mastered during the screw placement. Studies have shown that during sacroiliac screw placement, the incidence of screw placement errors is very high, even reaching more than 10%. Moreover, the study pointed out that the deviation of 4° during screw placement may cause sacrum canal nerve and blood vessel damage (26). However, with the assistance of navigation technology, a large number of shortcomings of sacroiliac screw surgery had been improved (11).

AIOOR had a dual fluoroscopic imaging system. The guide needle sleeve used in the AIOOR is made of entirely roentgenolucent PEEK material. When the X-ray image of the position is taken, there is no occlusion to the position of the screw, so that the surgeon could accurately understand the position of the pilot sleeve, which achieving completely unscreened and improving the accuracy of screw placement. During the operation, it could also realize the fine adjustment of the front-end sleeve device to further ensure the sacroiliac joint screw placement’s accuracy and safety. Our results showed that although the overall surgical time of the robot group was longer than that of the freehand group due to equipment placement, the actual screw placement time of the robot group was shorter than that of the free hand group, and the angle deviation of the screw was significantly different between the two groups.

The orthopaedic surgery robot assistant system inevitably has some limitations. Firstly, surgeons need to pay constant attention to the position of the optical tracker, and the photosensitive ball fixed on the patient and the robotic arm to avoid occlusion and affect the operation of the robotic arm. Secondly, the appilication of the orthopaedic surgery robot assistance system will increase surgical costs, such as the machine’s cost. However, it has the advantages of accuracy of screw placement, less radiographic exposure times to orthopaedic surgeons and patients, less blood loss and safety compared with the traditional freehand screw placement by orthopaedic surgeons. It is a new and more effective operation method, and it has outstanding application value and prospect.

Common fixation methods for sacral fractures are C-clamp, anterior sacral plate fixation, posterior sacral bolt fixation, sacral plate fixation, and sacral lag screw fixation. The selection of screws can be divided into partially threaded hollow screws, full-threaded hollow sacroiliac screws and solid screws, while solid screws are rarely used because they are difficult to close percutaneous placement and cannot be closed percutaneous removal. Postoperative loosening and nailing make the sacroiliac screw less stable in the vertical direction, which may lead to internal fixation failure, especially in osteoporosis. Our experience is that for senile sacral fractures, if full screw placement is not possible, a partially threaded compression screw is used, with a pad at the end of the screw to prevent the end of the screw from penetrating the iliac cortex.

Our next steps are to expand the number of cases in the study, along with long-term follow-up of the cases, to study the long-term benefits of the surgery, and to conduct a more comprehensive and accurate evaluation of the surgical effectiveness of robot-assisted screw fixation in the treatment of pelvic fractures.

## Data availability statement

The original contributions presented in the study are included in the article/supplementary material, further inquiries can be directed to the corresponding author.

## Ethics statement

The studies involving humans were approved by Ethics Committee of Inner Mongolia Medical University Affiliated Hospital. The studies were conducted in accordance with the local legislation and institutional requirements. The participants provided their written informed consent to participate in this study. Written informed consent was obtained from the individual(s) for the publication of any potentially identifiable images or data included in this article.

## Author contributions

Y-zZ for design and writing. X-dH for clinical evaluation. S-bW participated in the implementation of the robot. GL for clinical application. All authors contributed to the article and approved the submitted version.
